# The role of energy deficit in autophagy failure in Parkinson’s disease

**DOI:** 10.3389/fnagi.2026.1846307

**Published:** 2026-06-03

**Authors:** Mihajlo Bosnjak, Maja Misirkic Marjanovic, Milica Kosic, Milos Mandic, Ljubica Vucicevic, Verica Paunovic, Ljubica Harhaji-Trajkovic

**Affiliations:** 1 Faculty of Medicine, Institute of Microbiology and Immunology, University of Belgrade, Belgrade, Serbia; 2 Department of Neurophysiology, Institute for Biological Research “Sinisa Stankovic”-National Institute of Republic of Serbia, University of Belgrade, Belgrade, Serbia

**Keywords:** ATP depletion, autophagy, mitochondrial dysfunction, mitophagy, neurodegeneration, Parkinson’s disease

## Abstract

Mitochondrial complex I dysfunction, ATP depletion, and impaired autophagy are key features of Parkinson’s disease (PD), but their causal relationship remains unclear. Although energy stress induces autophagy, autophagy execution requires ATP. Available evidence suggests a biphasic effect of ATP depletion on autophagy in PD, with mild early energy decline promoting autophagy and more severe ATP loss, below a critical threshold, suppressing its completion. This mechanism may contribute to the accumulation of dysfunctional mitochondria and other undegraded cargo, creating a vicious cycle in which mitochondrial dysfunction, ATP decline, and autophagy failure progressively reinforce one another in PD. Here, we review current evidence linking cellular energy status to autophagic dysfunction in PD and discuss its pathogenic and therapeutic implications.

## 1 Introduction

Parkinson’s disease (PD) is a progressive neurodegenerative disorder characterized by the selective degeneration of dopaminergic neurons in the substantia nigra pars compacta. It is the second most common neurodegenerative disease worldwide, and its prevalence increases markedly with age. PD manifests through motor symptoms such as tremor, rigidity, bradykinesia and postural instability, accompanied by non-motor symptoms including cognitive decline, sleep disturbances and autonomic dysfunction. Despite decades of research, current treatments for PD remain largely symptomatic and do not halt the progressive loss of dopaminergic neurons (Ascherio and Schwarzschild, 2016; Jankovic and Tan, 2020).

At the cellular level, PD is characterized by mitochondrial dysfunction, oxidative stress, accumulation of misfolded α-synuclein, impairment of the autophagy-lysosomal system, neuroinflammation, and defects in protein quality control and vesicular trafficking (Jankovic and Tan, 2020). Among these interconnected processes, mitochondrial dysfunction has emerged as a central pathogenic event. It includes structural and morphological mitochondrial abnormalities, and impaired respiratory-chain activity, particularly complex I-dependent oxidative phosphorylation (OXPHOS), reduced mitochondrial membrane potential, compromised ATP synthesis, increased mitochondrial ROS production, mtDNA damage, altered calcium handling, disturbed mitochondrial dynamics and transport, and defective mitochondrial quality control through mitophagy (Flønes and Toker, 2024; Lucchesi and Biso, 2025).

Autophagy maintains neuronal homeostasis by removing damaged organelles and protein aggregates (Dikic and Elazar, 2018). It mediates the clearance of dysfunctional mitochondria via mitophagy and promotes the degradation of toxic α-synuclein species. Accordingly, autophagy failure may contribute to PD pathogenesis by allowing damaged mitochondria and aggregation-prone proteins to accumulate. Although pharmacological activation of autophagy has shown beneficial effects in experimental PD models (Pupyshev et al., 2019, 2021; Hebron et al., 2013; Hou et al., 2015; Xiong et al., 2011), clinical translation has been limited (Khan et al., 2024; Simuni et al., 2021; Guttuso et al., 2023).

**FIGURE 1:**
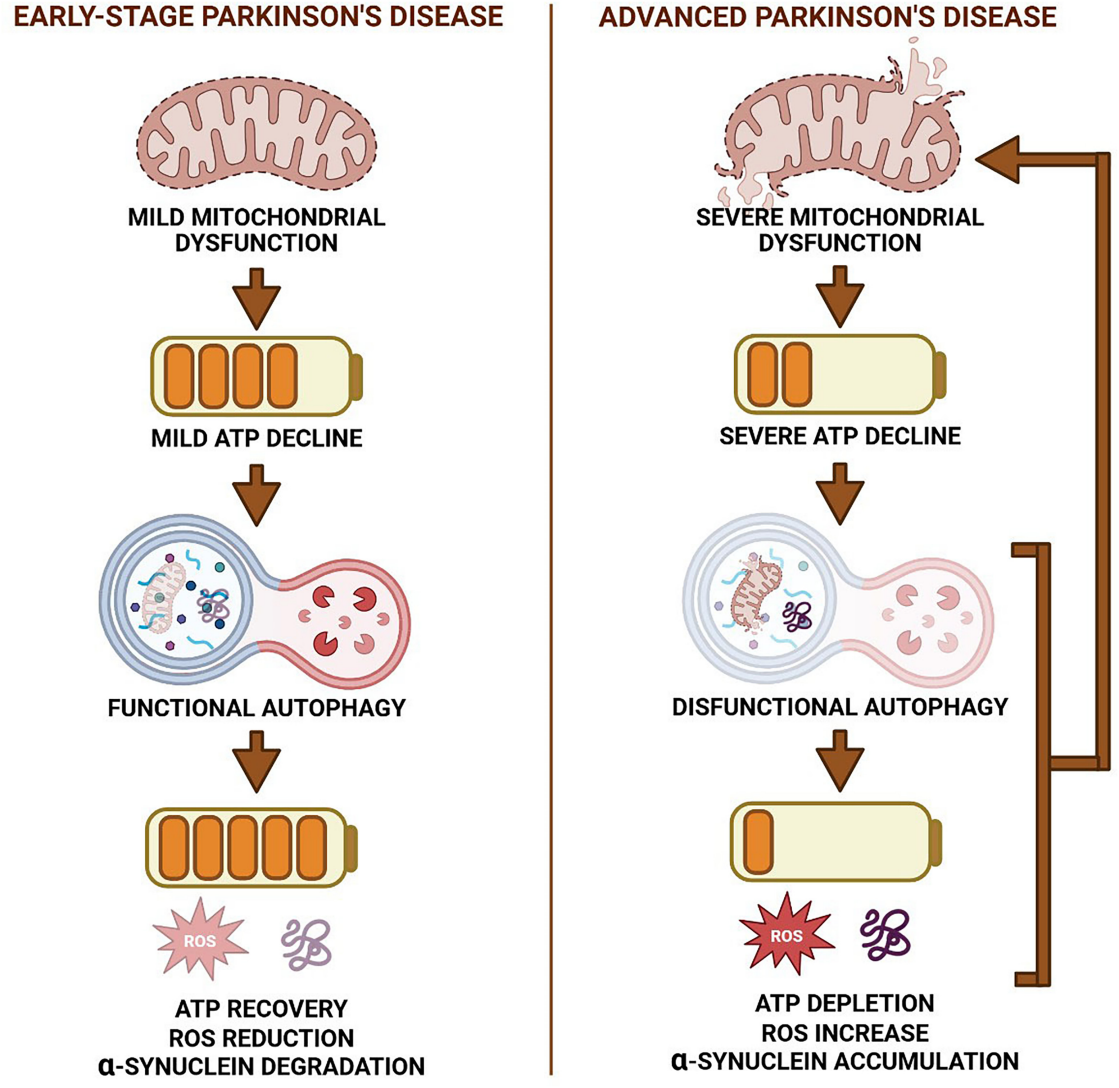
Hypothetical biphasic relationship between mitochondrial dysfunction, ATP availability and autophagy in Parkinson’s disease. In early-stage Parkinson’s disease, mild mitochondrial dysfunction may cause a moderate decline in ATP levels that is still compatible with functional autophagy. Under these conditions, autophagy/mitophagy may support cellular homeostasis by promoting damaged mitochondria removal, ATP recovery, ROS reduction and α-synuclein degradation. In advanced Parkinson’s disease, severe mitochondrial dysfunction may lead to profound ATP decline, thereby impairing ATP-dependent steps of autophagy. Dysfunctional autophagy may then contribute to incomplete cargo degradation, ATP depletion, increased ROS production and α-synuclein accumulation, creating a self-amplifying cycle that further promotes mitochondrial damage. ATP, adenosine triphosphate; ROS, reactive oxygen species. Created in BioRender. Todorovic, N. (2026) https://BioRender.com/azev5bq.

Energy metabolism and autophagy are tightly interconnected processes. By degrading damaged cellular components, autophagy supplies substrates that can re-enter metabolic pathways to support ATP production during cellular stress (Guo et al., 2016). In addition, mitophagy improves the overall quality of the mitochondrial network and enhances respiratory efficiency (Xiong et al., 2020). Accordingly, energy depletion is one of the major stimuli for autophagy induction. However, it is often overlooked that autophagy itself is an energy-dependent process (Mandic and Paunovic, 2024). This raises an important question in PD pathogenesis: whether moderate energy stress may activate adaptive autophagy and mitophagy, whereas sustained bioenergetic failure may suppress autophagic flux once a critical ATP threshold is reached. Under these conditions, impaired autophagy could further exacerbate bioenergetic collapse by promoting the accumulation of dysfunctional mitochondria and limiting substrate recycling, thereby creating a self-amplifying cycle that drives dopaminergic neurodegeneration. This hypothesis is outlined in Figure 1 and discussed in detail below.

## 2 Mitochondrial dysfunction and energetic failure in Parkinson’s disease

The principal energy substrate in the brain is glucose. It is metabolized through glycolysis, the tricarboxylic acid (TCA) cycle, and OXPHOS, which relies on the electron transport chain (complexes I-V) to sustain ATP production (Hall et al., 2012). Neurons require a continuous supply of energy to maintain essential cellular functions such as membrane potential, synaptic transmission, axonal transport, and intracellular trafficking (Chamberlain and Sheng, 2019). Dopaminergic neurons are particularly energy-demanding due to their extensive axonal arborization and continuous pacemaking activity, synaptic vesicle cycling, and long-distance axonal transport (Pissadaki and Bolam, 2013; Pacelli et al., 2015). Consequently, they are especially vulnerable to mitochondrial dysfunction and bioenergetic disturbances, which are considered key events in PD pathogenesis, as supported by extensive evidence (Pacelli et al., 2015).

Postmortem analyses of the substantia nigra from patients with PD revealed a marked reduction in mitochondrial complex I activity (Schapira et al., 1990; Flønes and Toker, 2024). The broader relevance of complex I dysfunction is supported by mitochondrial genetic disorders with parkinsonian or other movement-disorder features caused by mutations in mtDNA-encoded complex I subunit genes, such as *MT-ND1*, *MT-ND4* and *MT-ND6*, or in genes involved in mtDNA maintenance and homeostasis, such as *POLG* and *TFAM* (Davidzon et al., 2006; Nikoskelainen et al., 1995; Reinson and Õunap, 2020; Luoma et al., 2004; Balafkan et al., 2012; Gaweda-Walerych et al., 2010; Flønes and Toker, 2024). Magnetic resonance spectroscopy revealed reduced ATP levels in the putamen and midbrain of PD patients (Hattingen et al., 2009; Kudo et al., 1997). Bioenergetic impairment is not restricted to the brain, as spectroscopy analyses of skeletal muscles have shown reduced ATP production together with decreased nicotinamide adenine dinucleotide (NAD) levels in PD patients (Mischley et al., 2023). Moreover, aging, a major PD risk factor, is associated with an average 8% per decade decline in ATP-producing capacity (Payne and Chinnery, 2015). Neurotoxins 1-methyl-4-phenyl-1,2,3,6-tetrahydropyridine (MPTP), its active metabolite 1-methyl-4-phenylpyridinium (MPP^+^), and rotenone inhibit mitochondrial complex I *in vitro* and in vivo, while genetic models such as MitoPark mice recapitulate progressive parkinsonism due to selective mitochondrial respiratory chain deficiency (Smeyne and Jackson-Lewis, 2005; Xiong et al., 2012; Ekstrand et al., 2007). ATP-boosting agents, including creatine, acetyl-L-carnitine, terazosin, nicotinamide mononucleotide (NMN) and nicotinamide riboside (NR), have been reported to protect dopaminergic neurons and improve motor or cognitive phenotypes mainly by enhancing cellular energy metabolism, but also by reducing neuroinflammation, while their ability to decrease α-synuclein accumulation may be related to improved autophagy-dependent proteostatic clearance (Beal, 2011; Burks et al., 2019; Sarkar et al., 2015; Cai et al., 2019; Lu et al., 2014; Schöndorf et al., 2018; The Ninds Net-Pd Investigators, 2006; Yulug et al., 2025).

## 3 Autophagy and its energy-dependence

Neuronal proteostasis is maintained mainly by two complementary degradative systems: the ubiquitin–proteasome system (UPS) and autophagy. While the UPS primarily degrades short-lived and soluble misfolded proteins, autophagy is a lysosomal degradation pathway responsible for the removal of long-lived proteins, protein aggregates and damaged organelles (Passi et al., 2026). Because neurons cannot dilute damaged cellular components through cell division, efficient protein and organelle quality control is essential for neuronal homeostasis and survival (Stavoe and Holzbaur, 2019), while dysfunction of either pathway contributes to neurodegeneration (Passi et al., 2026).

Autophagy comprises three main forms: macroautophagy, chaperone-mediated autophagy (CMA), and microautophagy, which differ in how cytoplasmic cargo reaches lysosomes (Parzych and Klionsky, 2014). Microautophagy involves direct lysosomal engulfment of cargo, whereas CMA selectively delivers soluble proteins to lysosomes through heat shock cognate 70 kDa protein (HSC70)- and lysosome-associated membrane protein type 2A (LAMP2A)-dependent translocation (Oh et al., 2022). CMA is particularly relevant to PD because it contributes to the lysosomal degradation of α-synuclein (Cuervo et al., 2004).

In macroautophagy, cargo is sequestered within a growing phagophore that elongates and closes to form a double-membrane autophagosome, which subsequently fuses with lysosomes for cargo degradation and recycling, a dynamic process referred to as autophagic flux (Fleming et al., 2022; Parzych and Klionsky, 2014). Within macroautophagy, mitophagy and aggrephagy are particularly relevant to PD, as they mediate the clearance of dysfunctional mitochondria and toxic protein aggregates, including α-synuclein (Fleming et al., 2022). In PTEN-induced kinase 1 (PINK1)/Parkin-mediated mitophagy, mitochondrial depolarization stabilizes PINK1 on the outer mitochondrial membrane, leading to Parkin recruitment and ubiquitin-dependent labeling of damaged mitochondria for autophagosomal delivery to lysosomes. By sustaining mitochondrial quality control, mitophagy supports neuronal bioenergetics (Jin and Youle, 2012).

Mild or moderate energy and nutrient stress classically activates autophagy. At the signaling level, this response is mediated, at least in part, by AMP-activated protein kinase (AMPK), a central sensor of cellular energy status, which promotes autophagy through inhibition of mechanistic target of rapamycin complex 1 (mTORC1) and activation of the unc-51-like autophagy activating kinase 1 (ULK1) complex, whereas nutrient-rich phosphoinositide 3-kinase–Akt (PI3K–Akt)–mTORC1 signaling suppresses ULK1-dependent autophagy initiation (Kim et al., 2011; Egan et al., 2011). NAD^+^-dependent sirtuin 1 also links cellular metabolic and redox status to autophagy by regulating autophagy-related proteins and stress-responsive transcriptional programs (Lee et al., 2008).

However, this canonical view often overlooks an important point: autophagy is not only triggered by energy stress, but also requires energy for its execution (Mandic and Paunovic, 2024). Although autophagy is transcriptionally regulated and transcription/translation are energetically demanding processes (Conaway and Conaway, 1988; Hu and Dourado, 2020), acute autophagy induction during energy stress does not necessarily require *de novo* protein synthesis (Plomp et al., 1987). However, under sustained energy-depleting conditions, reduced expression of autophagy-related (ATG) genes may compromise autophagy capacity and contribute to impaired autophagic flux (Bosc et al., 2020; Yang et al., 2013; Xi et al., 2013; de Theije et al., 2018; Lee et al., 2015; Vucicevic et al., 2020). Energy is also required for the interactions of ATG proteins, including ATG5–ATG12, ATG3–LC3, and ATG7–ATG8 interactions, as well as LC3-I lipidation into autophagosome associated LC3-II (Ge et al., 2013; Maruyama and Noda, 2017; Shao et al., 2007; Yamaguchi et al., 2018; Taherbhoy et al., 2011). ATP hydrolysis supports key membrane-remodeling steps during autophagosome biogenesis, from omegasome constriction and phagophore expansion to final phagophore closure (Nähse and Raiborg, 2023; Melia et al., 2020). Fusion of autophagosomes with lysosomes also requires ATP (Koga et al., 2010; Nara et al., 2002). In addition, lysosomal acidification, maintained by the ATP-consuming vacuolar H^+^-ATPase, is essential for autophagosome–lysosome fusion (Kawai et al., 2007), as well as for lysosomal hydrolase activity and cargo degradation (Mindell, 2012). Some authors have suggested that autophagy becomes compromised when ATP levels decline by approximately 50% (Xi et al., 2013), but the precise energetic threshold remains uncertain and likely varies depending on cell type, metabolic state, substrate availability, and duration of stress.

From a bioenergetic perspective, autophagy can be regarded as an energetically costly but potentially beneficial resource allocation. Cells consume ATP to degrade intracellular constituents and recycle amino acids, fatty acids, sugars, and other metabolites that can subsequently support ATP production, while also removing damaged mitochondria and preserving mitochondrial quality, thereby sustaining cellular ATP-generating capacity. However, when ATP availability becomes critically limited, the energetic cost of sustaining autophagic flux may outweigh its delayed metabolic benefit (Mandic and Paunovic, 2024). Consistently, recent studies have shown that during severe glucose starvation or mitochondrial energy crisis, defined as profound mitochondrial ATP deficiency that cannot be adequately compensated by glycolysis, the central energy sensor AMPK, may paradoxically suppress ULK1-dependent autophagy initiation. This response is thought to preserve autophagy machinery from caspase-mediated degradation, thereby enabling rapid recovery of autophagy once energy balance is restored (Park et al., 2023; Kim, 2024; Mandic and Misirkic Marjanovic, 2025). Moreover, mitophagy could become disadvantageous during profound energy deprivation, since partially damaged mitochondria may retain residual ATP-producing capacity (Li et al., 2022).

## 4 Autophagy impairment in Parkinson’s disease

Accumulating evidence from human tissue, patient-derived neurons, genetic studies, and toxin-based models indicates that both macroautophagy and CMA are impaired in PD. Analyses of postmortem brain samples have revealed accumulation of autophagic vacuoles together with lysosomal depletion, indicating impaired autophagosome clearance rather than efficient autophagic flux (Dehay et al., 2010). Midbrain neurons derived from PD patient-induced pluripotent stem cells display impaired autophagosome-lysosome fusion (Pitcairn et al., 2023). In postmortem PD brains, levels of the CMA markers LAMP2A and HSC70 are reduced in the substantia nigra, consistent with defective lysosomal protein clearance (Alvarez-Erviti et al., 2010). Several PD-associated genes encode proteins that converge on autophagy–lysosomal dysfunction. These include *SNCA*, which encodes α-synuclein, whose mutant or aggregated forms impair autophagic clearance, *GBA1*, which encodes glucocerebrosidase, and *ATP13A2*, which supports lysosomal degradation and homeostasis, *LRRK2*, which regulates autophagosome–lysosome trafficking and lysosomal function, *PINK1* and *PRKN*, which encode PINK1 and Parkin, respectively, and mediate mitophagy, and *PARK7*, which encodes DJ-1 and supports mitochondrial stress responses and mitophagy (Erekat, 2022). Finally, suppressed autophagic flux has been demonstrated in multiple neurotoxin-based PD models, including MPTP/MPP+, rotenone, and 6-hydroxydopamine (6-OHDA) in which autophagosome accumulation is accompanied by lysosomal dysfunction and defective cargo clearance (Dehay et al., 2010; Miyara et al., 2016; He et al., 2018; Wu et al., 2015).

Before considering ATP depletion as a potential contributor to autophagy failure in PD, it is important to emphasize that autophagy dysfunction in this disease is multifactorial and can arise from several mechanisms. Pathological accumulation of α-synuclein interferes with multiple stages of autophagy. α-synuclein inhibits autophagosome biogenesis by disrupting ATG9 recruitment to the omegasome (Winslow et al., 2010). Aggregated α-synuclein can also impair autophagosome-lysosome fusion, leading to defective cargo clearance (Tang et al., 2021). Mutant or dopamine-modified α-synuclein binds to LAMP2A thereby blocking lysosomal translocation of other CMA substrates (Cuervo et al., 2004; Martinez-Vicente et al., 2008). Oxidative and nitrosative stress, both prominent features of PD pathology, may further compromise autophagy. Although moderate oxidative stress initially stimulates autophagy, excessive ROS and reactive nitrogen species can damage the autophagic machinery itself. For example, S-nitrosylation of p62/SQSTM1, a selective autophagy receptor that links ubiquitinated cargo to the autophagosomal membrane, inhibits autophagic flux (Oh et al., 2022). Moreover, prolonged oxidative stress induced by the parkinsonian toxin paraquat inhibits autophagosome formation (Janda et al., 2015). Nitrosative stress also suppresses mitophagy through S-nitrosylation of PINK1 (Oh et al., 2017). PD-linked genetic defects may also impair the autophagy-lysosome pathway. Pathogenic *LRRK2* mutations disrupt phagophore biogenesis, autophagosome formation, autophagosome-lysosome fusion, and lysosomal function (Madureira et al., 2020). Mutations in *GBA1* reduce the activity of lysosomal hydrolase glucocerebrosidase, leading to lysosomal dysfunction and α-synuclein accumulation (Bougea, 2025). Mutations in *ATP13A2*, a lysosomal P-type ATPase, impair lysosomal acidification and thereby block the terminal degradative step of autophagy (Ramirez et al., 2006). Likewise, *VPS35* mutations impair LAMP2A recycling and reduce lysosomal protein clearance (Tang and Erion, 2015). Aberrant mTOR signaling also contributes to autophagy suppression in PD. Impaired macroautophagy in an A53T α-synuclein cellular model was accompanied by increased mTOR/70-kDa ribosomal protein S6 kinase (p70S6K) signaling (Jiang et al., 2013). A recent review summarized evidence from cellular and animal PD models showing that several microRNAs, small non-coding RNAs that silence target mRNAs, regulate autophagy-related pathways (Ma et al., 2023). For example, miR-124 is downregulated in MPTP-treated mice and MPP^+^-intoxicated SH-SY5Y cells, and its restoration improves impaired autophagy and reduces dopaminergic neuronal loss, partly by targeting Bim (Wang et al., 2016). miR-181b is downregulated in MPP^+^-treated PC12 cells, and its overexpression inhibits excessive autophagy and improves cell viability by suppressing PTEN and activating the Akt/mTOR pathway (Li et al., 2018).

Thus, autophagy dysfunction in PD can be attributed to pathological α-synuclein accumulation, oxidative and nitrosative stress, PD-linked genetic mutations, dysregulated intracellular signaling pathways, and post-transcriptional regulation. These established pathways may directly impair autophagic flux and may also intersect with each other, as well as with mitochondrial dysfunction and cellular energy metabolism. Indeed, α-synuclein accumulation, oxidative/nitrosative stress, and PD-linked mutations in *PINK1*/*PRKN*, *LRRK2*, *GBA1*, *ATP13A2*, or *PARK7* have all been associated with impaired mitochondrial quality control, respiratory-chain dysfunction, reduced mitochondrial membrane potential, oxidative damage, and/or ATP depletion (Mullin and Schapira, 2013; Subramaniam and Chesselet, 2013; Cleeter et al., 2013; Park et al., 2014; Singh and Prescott, 2021; Gonzalez-Hunt et al., 2020).

## 5 Energy depletion may inhibit autophagy in Parkinson’s disease

**FIGURE 2:**
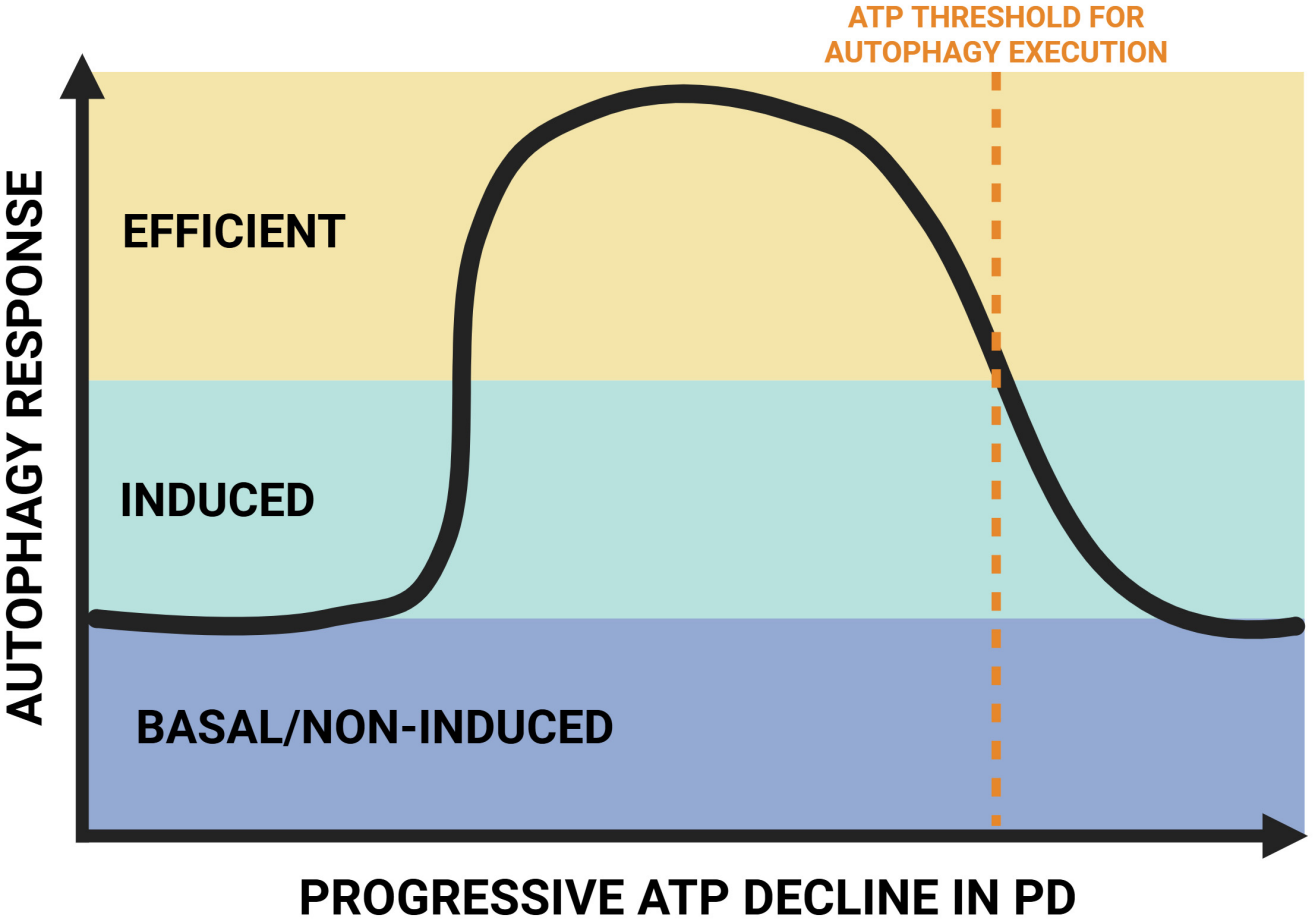
Conceptual framework for the ATP dependence of the autophagic response in Parkinson’s disease. Progressive ATP decline in PD initially promotes autophagy induction, and within a permissive energetic range, autophagy can proceed efficiently. However, when ATP levels fall below a critical threshold required for effective autophagy execution, the autophagic response may become abortive, with autophagy being induced but not efficiently completed, or may fail to be induced altogether under conditions of more severe energy depletion. ATP, adenosine triphosphate; PD, Parkinson’s disease. Created in BioRender. Todorović, N. (2026) https://BioRender.com/kiv2vpc.

Given that autophagy requires energy and PD is marked by mitochondrial dysfunction and bioenergetic deficits, energy depletion itself may impair autophagy execution. In the early stages of PD, mild mitochondrial dysfunction and limited energy depletion may stimulate, or at least permit a productive autophagic response. With disease progression, however, cumulative mitochondrial damage and progressive ATP loss may increasingly compromise energy-dependent steps of the autophagic pathway. Consequently, autophagy may become interrupted at progressively earlier stages, shifting from a functional, protective response to partial, and eventually autophagy failure (Figure 2).

Evidence from neurotoxin-based PD models provides the most direct support for a link between mitochondrial inhibition, ATP depletion and autophagy impairment. In SH-SY5Y neuroblastoma cells, the complex I inhibitor rotenone caused ATP depletion accompanied by impaired lysosomal acidification and reduced degradation of p62 and α-synuclein aggregates, indicating inhibition of autophagic flux at the level of lysosomal degradation. Notably, these effects were associated with increased lysosomal pH, consistent with the energy dependence of lysosomal acidification. The progressive ATP decline from 6 to 24 h correlated with stronger autophagic blockade, suggesting that worsening bioenergetic stress aggravates autophagy impairment (Mader et al., 2012). Time-dependent effects were also reported in SH-SY5Y cells, where rotenone-induced inhibition of OXPHOS and ATP depletion increased levels of the lipidated autophagosome marker microtubule-associated protein 1 light chain 3-II (LC3-II) after 72 h but decreased LC3-II after prolonged exposure (96 h), suggesting that more severe or sustained bioenergetic stress may suppress autophagosome formation. However, without direct assessment of autophagic flux, it cannot be determined whether reduced LC3-II levels reflect impaired LC3-I lipidation/autophagosome formation or enhanced autolysosomal degradation (Damri et al., 2021). In SH-SY5Y cells, mild prolonged MPP+ exposure caused progressive depletion of extracellular glucose, consistent with increased glycolytic consumption after complex I inhibition. This was accompanied by an initial enhancement of autophagosome synthesis followed by impaired autolysosomal degradation, and the reversal of these changes by glucose supplementation supports a contribution of bioenergetic insufficiency to autophagic flux failure (Sakamoto et al., 2017). Likewise, pyruvate restored mitochondrial complex I activity and stimulated mitophagy in MPP+-treated dopaminergic neurons. The authors concluded that this effect was due to PINK-1 stabilization on damaged mitochondria and independent of OXPHOS and cellular ATP levels. However, because this interpretation was based mainly on experiments using mitochondrial uncoupler carbonyl cyanide m-chlorophenyl hydrazone (CCCP), a contribution of bioenergetic improvement under MPP+ treatment cannot be excluded (Park et al., 2015). In addition, numerous studies have reported that MPP^+^ and rotenone inhibit autophagy without directly assessing cellular ATP levels, leaving the contribution of energy depletion unresolved (Miyara et al., 2016; Cai et al., 2009; Zhang et al., 2017; Azam and Haque, 2022; Bai et al., 2025; Prasertsuksri et al., 2023; Li et al., 2020; Xiong et al., 2013; Shirgadwar et al., 2023; Zeng et al., 2019; Arduíno et al., 2012; Jovanovic-Tucovic et al., 2019). Given that both toxins inhibit mitochondrial complex I, ATP depletion is plausible but cannot be assumed with certainty, because compensatory glycolysis may partially offset the bioenergetic deficit (Dranka et al., 2012).

Evidence from genetic, familial and α-synuclein-related PD models also supports a potential interaction between cellular bioenergetics and autophagy-related clearance mechanisms. Terazosin, an activator of the glycolytic enzyme phosphoglycerate kinase 1, increased intracellular ATP levels and promoted autophagy-associated clearance of pathological protein aggregates in neurodegeneration models, including a rat α-synucleinopathy model induced by viral α-synuclein expression in the striatum (Chen et al., 2023). Moreover, lactate and pyruvate activated autophagy and mitophagy and protected cells in both MPP+-based toxic PD models (Fedotova and Dolgacheva, 2022) and familial PD fibroblast models carrying *PINK1/PARK2* or *SNCA*-related mutations (Komilova and Angelova, 2022; Fedotova and Dolgacheva, 2022). The authors attributed these effects primarily to transient intracellular acidification, and the studies did not directly examine an ATP-mediated mechanism. Nevertheless, because lactate and pyruvate can feed into TCA cycle and thereby support mitochondrial energy metabolism, a contribution of cellular bioenergetic changes cannot be excluded.

Although complex I deficiency is the best-documented respiratory-chain defect in PD, other OXPHOS components have also been reported to be suppressed (Subrahmanian and LaVoie, 2021). Therefore, studies outside classical PD models in which mitochondrial energy synthesis was experimentally disrupted at different levels may provide useful mechanistic insight into how bioenergetic failure can suppress autophagy. Early work in amino acid-starved kidney tubules showed that ATP depletion induced by anoxia, oligomycin-mediated ATP synthase inhibition or dinitrophenol-induced mitochondrial uncoupling all reduced autophagic vesicle formation (Shelburne et al., 1973). Subsequent studies in hepatocytes showed that ATP depletion induced by anoxia, dinitrophenol or atractyloside-mediated blockade of mitochondrial ADP/ATP exchange impaired autophagic sequestration, altered lysosomal acidification and decreased autophagic proteolysis (Plomp et al., 1989; Plomp et al., 1987; Schellens and Meijer, 1991; Schellens et al., 1988, 1990). In more recent studies, metformin-mediated complex I inhibition and antimycin A-mediated complex III inhibition reduced ATP levels and suppressed basal autophagic flux in leukemia cells, as shown by decreased LC3 puncta, LC3-II levels and lipid degradation, in part through disruption of mitochondria–endoplasmic reticulum contact sites required for autophagosome biogenesis (Thomas et al., 2018). Similarly, phenformin, metformin, rotenone, oligomycin A and genetic complex I disruption reduced ATP availability and impaired mTOR inhibition-induced autophagy in kidney-, liver-, lung- and cardiomyocyte-derived cell lines, mainly by suppressing autophagosome formation rather than lysosomal acidification (Bosc et al., 2020). Genetic defects affecting mitochondrial respiratory-chain function provide additional support. In fibroblasts from patients with mitochondrial diseases, including mitochondrial encephalomyopathy, lactic acidosis, and stroke-like episodes (MELAS) and other respiratory-chain defects, reduced mitochondrial ATP production was associated with abnormal accumulation of autophagic/lysosomal compartments, impaired autophagosome clearance, or reduced Beclin 1 expression and LC3 conversion, indicating defective autophagic flux (Cotán et al., 2011; Morán et al., 2014; Deng et al., 2020). Similarly, Leber’s hereditary optic neuropathy (LHON)-associated complex I mutations were accompanied by reduced mitochondrial ATP generation and impaired PINK1/Parkin-mediated mitophagy, together with broader autophagy–lysosomal defects, including reduced LC3-II levels, impaired p62 degradation and lower LAMP1 expression (Liang et al., 2022; Zhang et al., 2021).

Importantly, the link between ATP depletion and autophagy inhibition is supported by studies showing a positive correlation between intracellular ATP levels and the magnitude of autophagic flux (Plomp et al., 1987; Plomp et al., 1989; Schellens et al., 1988; Schellens and Meijer, 1991) and that restoring cellular bioenergetics can attenuate autophagy impairment (Plomp et al., 1987; Thomas et al., 2018). Taken together, findings from PD-relevant models and non-PD models in which OXPHOS or mitochondrial ATP production was experimentally impaired support the possibility that bioenergetic failure may compromise autophagy by limiting ATP-dependent steps, including autophagosome formation/biogenesis (Shelburne et al., 1973; Thomas et al., 2018; Bosc et al., 2020; Schellens and Meijer, 1991; Deng et al., 2020), autophagosome-mediated sequestration of cytoplasmic cargo (Plomp et al., 1987; Plomp et al., 1989; Schellens et al., 1988, 1990), lysosomal acidification (Mader et al., 2012; Deng et al., 2020; Plomp et al., 1987, 1989; Schellens and Meijer, 1991), autophagosome–lysosome fusion (Cotán et al., 2011; Morán et al., 2014), and cargo degradation/proteolysis (Thomas et al., 2018; Bosc et al., 2020; Mader et al., 2012; Cotán et al., 2011; Morán et al., 2014; Plomp et al., 1987, 1989; Schellens et al., 1990; Schellens and Meijer, 1991; Sakamoto et al., 2017; Chen et al., 2023).

## 6 Discussion

### 6.1 Implications

Because energy depletion is classically viewed as a trigger of autophagy, the possibility that severe energy deficit may instead suppress autophagy remains largely underexplored. Beyond a certain threshold of bioenergetic decline, autophagy can no longer be efficiently initiated or completed in PD because ATP availability becomes insufficient to support its execution (Mandic and Paunovic, 2024). The extent of energy depletion may be a critical determinant of whether autophagy is activated or suppressed (Figure 2). However, it remains difficult to define the precise level of ATP depletion at which this shift occurs. A reduction of cellular ATP by approximately one half has previously been proposed as a threshold for autophagy inhibition, as observed in hypoxic cancer cells treated with 2-deoxyglucose or subjected to glucose starvation (Xi et al., 2013). However, numerous aforementioned studies indicate that autophagy impairment can occur under more modest ATP depletion (Xi et al., 2013; Plomp et al., 1987, 1989; Schellens et al., 1988; Schellens and Meijer, 1991; Schütt et al., 2012; Damri et al., 2021; Bosc et al., 2020; Thomas et al., 2018; Morán et al., 2014; Cotán et al., 2011; Liang et al., 2022; Zhang et al., 2021). These findings suggest that the sensitivity of autophagy to energy decline depends on cellular context and metabolic state rather than on a fixed ATP threshold. Graded ATP depletion may explain why complex I inhibitors can either induce or suppress autophagy, with suppression prevailing once ATP falls below a context-dependent threshold.

Incomplete autophagy may be especially harmful, as impaired autophagosome clearance promotes accumulation of non-functional vacuoles and inefficient cargo degradation, including α-synuclein, instead of productive recycling (Sakamoto et al., 2017; Button et al., 2017). Under such conditions, ATP is consumed without generating the expected bioenergetic benefit of cargo recycling and mitochondrial quality control through mitophagy (Dikic and Elazar, 2018; Xiong et al., 2020). Primary ATP depletion may therefore, through inhibition or incomplete execution of autophagy, lead to a secondary and progressive decline in cellular energy status, establishing a self-amplifying cycle. This bioenergetic deterioration may be further amplified by the fact that impaired autophagy also promotes oxidative stress and α-synuclein accumulation, both of which can further damage mitochondria and exacerbate energy failure (Xiao et al., 2022; Pukaß et al., 2015; Parihar et al., 2008; Figure 1).

This concept has important therapeutic implications. Despite beneficial effects of autophagy-inducing interventions in preclinical PD studies, including *in vivo* models (Pupyshev et al., 2019; Hebron et al., 2013), their clinical translation remains limited by the poor selectivity and pleiotropic actions of available autophagy modulators, limited brain exposure, uncertain autophagy target engagement in patients, and disease-stage heterogeneity (Khan et al., 2024; Simuni et al., 2021; Guttuso et al., 2023; Stevanovic et al., 2024). In addition to these limitations, the present framework suggests that autophagy stimulation may be ineffective when cellular energy availability is insufficient to support ATP-dependent steps of autophagy execution, highlighting the potential need to combine autophagy activation with restoration of neuronal bioenergetic capacity.

### 6.2 Future directions

Demonstrating that severe energy depletion directly contributes to autophagy inhibition in PD remains challenging because autophagy in diseased neurons is simultaneously influenced by α-synuclein accumulation, oxidative and nitrosative stress, lysosomal dysfunction, and PD-associated genetic alterations (Winslow et al., 2010; Cuervo et al., 2004; Martinez-Vicente et al., 2008; Tang et al., 2021; Oh et al., 2022, 2017; Janda et al., 2015; Madureira et al., 2020; Ramirez et al., 2006). These factors likely converge on common nodes of the autophagic pathway, making the specific contribution of ATP depletion difficult to isolate experimentally. Addressing this problem will require integrated experimental and computational approaches. Future studies should quantify, in parallel, ATP levels, mitochondrial function, oxidative stress, α-synuclein burden, lysosomal activity, and autophagic flux, and incorporate these variables into mathematical models capable of estimating their relative contribution to autophagy failure in PD. A key test of this model will be to determine whether metabolic interventions that restore cellular energy status can also restore autophagic flux. It will also be important to determine whether increasing bioenergetic stress shifts autophagy inhibition from late degradative steps to earlier stages such as autophagosome formation.

This model supports therapeutically combining energy enhancers with pharmacological autophagy activators to restore autophagic flux and promote neuroprotection in PD. Candidate metabolic interventions include agents that enhance cellular bioenergetics through distinct mechanisms, such as creatine, NR, NMN, pyruvate, ketone-based interventions, acetyl-L-carnitine, or terazosin. These could be evaluated in combination with mechanistically distinct autophagy activators, including mTOR inhibitors (rapamycin), mTOR-independent inducers (trehalose, lithium, spermidine), SIRT1/AMPK-linked modulators (resveratrol), or lysosome-targeting approaches (ambroxol), to determine whether simultaneous correction of bioenergetic failure and autophagy insufficiency provides superior protection compared with either strategy alone.

Given the multifactorial nature of PD pathogenesis, such combinatorial approaches may also need to be integrated with additional interventions targeting oxidative stress and other pathogenic processes. However, whether such strategies can provide meaningful benefit at advanced disease stages, when substantial neuronal loss has already occurred, remains uncertain. This raises the possibility that bioenergetic interventions may be most effective when applied early in disease progression, potentially at prodromal or other high-risk stages, before substantial neuronal loss develops.
